# Accounting for legacy nitrogen stores does not improve the accuracy of riverine nitrate load model predictions

**DOI:** 10.1088/3033-4942/ae09d6

**Published:** 2025-10-10

**Authors:** James N Carleton, Robert D Sabo

**Affiliations:** Center for Public Health and Environmental Assessment, Office of Research and Development, U.S. Environmental Protection Agency (Mail Code 8623R), 1200 Pennsylvania Ave NW, Washington, DC 20460, United States of America

**Keywords:** nitrogen yield, rivers, legacy nutrients, time lag

## Abstract

Legacy stores of nitrogen (N) have been proposed as an explanation for time lags between implementation of nutrient management practices and discernable declines in riverine N loads, particularly in agricultural watersheds. Process models that treat the environment as being divided into linked homogeneous pools or compartments have been used recently to estimate legacy N stores and infer time lags. However, questions have been raised regarding equifinality, i.e. the idea that acceptable estimates might be obtained using models with different structures and/or sets of parameters, creating uncertainty in model interpretation. To further advance understanding of legacy stores and lags in watershed N delivery, we developed six fate and transport models that vary in simplicity and physicochemical detail. Using published annual N surplus and riverine N load datasets, we calibrated the models to minimize summed squared errors compared with multidecadal NO_3_–N (nitrate N) yield time series at 254 riverine sites across the continental United States. All models fit most datasets well, with calibration results suggesting little if any lagged contribution from either biogeochemical or hydrologic legacy stores. The highest quality model in terms of adjusted Akaike information criterion was the simplest, comprising a single, well-mixed compartment that lacks any mechanism to represent time lags between N input and output. Calibrated model results thus fail to provide evidence of influential legacy N stores. However, results using incrementally lagged watershed N inputs (themselves shown to be temporally autocorrelated), also imply that delivery lags of up to about a decade cannot be ruled out.

## 1. Introduction

The chemistry of fresh waters is strongly influenced by watershed land uses and human activities, and the deliberate or accidental release into the environment of anthropogenically-derived substances, including nutrients (i.e. nitrogen [N] and phosphorus [P]), metals, and xenobiotic chemicals such as pesticides (e.g. Van Metre et al 2017, Chow et al 2020, Piwowarska and Kiedrzynska 2022, Oates et al 2025). Both urban and agricultural development have long been associated with increased nutrient concentrations in surface and groundwaters, resulting in nutrient loads delivered to downstream waters by streams and rivers (e.g. Dubrovsky et al 2010). Elevated nutrient concentrations are generally associated with deleterious effects on estuaries and other downstream waters because they stimulate the growth of algae, which often include toxin-releasing cyanobacteria. Algal decomposition draws oxygen from the water column, potentially leading to low oxygen ‘dead zones’ (Rabelais *et al* 2002, Dodds 2006, Wurtsbaugh et al 2019). Developing an accurate quantitative understanding of mechanisms that relate surface water nutrient concentrations and loads to their anthropogenic sources at the watershed scale is thus important for developing effective management actions to mitigate impacts on water quality and human and ecological health.

Although a complete understanding of mechanisms driving nutrient loads at watershed scales remains elusive, published results in recent years hint at the possibility of two contrasting paradigms relating nutrient export to hydrology (i.e. discharge) and watershed N inputs, for annual or longer time scales. Because national-scale P landscape input datasets (analogous to those we used to model N in this study) are not yet publicly available, this paper focuses specifically on N. Given differences between N and P in terms of mobility/sorption and loss/decay processes as well as sources to surface water (e.g. Alexander et al 2008, Manning et al 2020), these same paradigms may or may not be relevant for P.

Under the first paradigm, riverine nutrient load dynamics are dominantly controlled by (proportional to) discharge dynamics, as discharge variability greatly exceeds concentration variability. Concentrations are thus said to behave ‘chemostatically’ (Basu et al 2010, Zhang 2018), a phrase first coined by Godsey et al (2009) to describe observed concentration-discharge relationships (or lack thereof) for ionic mineral solutes. Various authors have characterized chemostatic behavior in terms of the ratio of the coefficients of variation of concentration and discharge (CV_*C*_/CV_*Q*_), and/or the slope (‘*b*’) of the ln(*C*) vs ln(*Q*) relationship, generally associating chemostatic behavior with values of both quantities near zero, and ‘mobilization’ and ‘dilution’ behavior with larger values of CV_*C*_/CV_*Q*_, and larger and smaller values of *b*, respectively (Thompson et al 2011, Musolff et al 2015, Ali et al 2017, Ehrhardt et al 2019).

Under the second paradigm, riverine nutrient loads are also proportional to discharge, but to contemporaneous watershed nutrient inputs as well—that is, they are proportional to the product of these two quantities. This is implied by the results of studies that have found riverine nutrient loads, when normalized by watershed surplus nutrient inputs, to be proportional to discharge (e.g. Caraco and Cole 1999, Howarth et al 2006, Schaefer et al 2009, Sobota et al 2009, Dupas et al 2015, Compton et al 2020). This second paradigm, which can be thought of as a variant on the first, implies that nutrient concentrations, rather than being essentially fixed, vary with changes in watershed nutrient inputs, though not with changes in discharge. The second paradigm may be difficult to distinguish from the first if temporal variations in nutrient inputs are small compared with those for discharge. This second paradigm also should not be confused with ‘chemodynamic’ behavior, wherein concentrations (rather than loads) vary as a function of discharge—a phenomenon that has been typically observed over shorter (i.e. sub-annual) time scales (Musolff et al 2015, Wan et al 2017, Marinos et al 2020).

To explain the chemostatic behavior observed for total N (TN) and total P (TP) in rivers draining agricultural watersheds, Basu et al (2010) suggested the presence of anthropogenic ‘legacies’ of accumulated nutrients resulting from years of watershed inputs that exceeded loss rates. Basu et al (2011) suggested that for N, these legacies comprise organic stores accumulated in agricultural soils, which release soluble forms of N into percolating soil water as soil organic N (SON) is gradually metabolized (i.e. ‘biogeochemical’ legacies). Other authors (e.g. Erhardt et al 2019, Guillaumot et al 2021, van Meter et al 2023) have suggested that N moving relatively slowly through groundwater may contribute to lagged riverine loads via direct discharge to surface water, e.g. in baseflow (i.e. ‘hydrologic’ legacies).

In this analysis, we define ‘lagged’ delivery as that in which N takes longer than 1 year to reach surface water following application to the landscape. We similarly define a ‘legacy store’ in this study simply as a pathway or collection thereof in which N resides longer than 1 year during its transport from landscape to surface water. We employ this definition because the watershed N input data (TREND, discussed below) we used in our modeling are provided in annual increments; thus, the models operate on a 1 year time step and lags of less than 1 year cannot be represented (this limitation also holds for the ELEMeNT model, discussed below). Lagged delivery does not contribute to riverine N export under either of the two mentioned paradigms (only same-year discharge and N inputs are considered). Nonetheless, while superficially similar, the two paradigms seem, if ‘true’, to imply very different watershed-scale transport mechanisms for N. Chemostatic behavior suggests an analogy for N with a dissolved-phase material (e.g. mineral solute) at chemical saturation and therefore fixed concentration, in equilibrium with solid-phase material akin to a legacy store. Concentrations that are fixed in magnitude provide no information about how large such a non-dissolved store might be, or how it might influence loads or concentrations if inputs that contribute to it were to decline. In contrast, concentrations under the second paradigm are proportional to concurrent watershed N input loads, and therefore not functions of input loads from previous years. In this case, rather than controlling riverine loads, legacy stores exert no discernable effect on loads even if they are present in the watershed.

Intermediate to the two paradigms sits the concept of depletable legacies, created by years of accumulated net N surplus, that influence concentration in a manner that mimics neither chemical saturation nor proportionality with same-year watershed N inputs. The presence of such legacy stores could create quantifiable time lags between watershed N inputs and riverine N delivery. Studies have documented lags of this sort between current-year riverine N export and prior year(s) watershed N inputs (e.g. Chen et al 2014, Dupas et al 2020, Stackpoole et al 2021), in apparent refutation of both the above-discussed paradigms (because lagged delivery, as we define it, is that which involves transport times of longer than 1 year and therefore does not contribute to riverine N export under either surface water N loading paradigm, as the paradigms relate annual loads either to same-year discharge solely, or to same-year discharge and same-year watershed N inputs). In most cases these documented lags were of less than about ten years in magnitude (i.e. current year N export is related to watershed N inputs 1–10 years earlier), although there have been exceptions. For example, Howden et al (2011) estimated a mean lag of 37 years between N-loading to the land and riverine NO_3_–N response in a small, rural UK watershed. Similarly, McIsaac et al (2001) and (2002) found that discharge in combination with mean net anthropogenic nitrogen input over prior year spans of 2–5 and 6–9 (as independent predictors) accounted for 95% of interannual variation in Mississippi River NO_3_–N yields (i.e. loads normalized by watershed area). In the case of P, McCrackin et al (2018) used a calibrated two-compartment, three-parameter model to estimate a 30 year transit time in catchments that drain to the Baltic Sea.

Intermediate situations, wherein legacies induce quantifiable lags in riverine delivery, can realistically only be simulated with dynamic mechanistic representations. Mechanistic fate and transport models and approaches that dynamically represent finite legacy nutrient stores are somewhat rare (Carleton and Lee 2023). A noteworthy exception is the ELEMeNT model, which has been used to estimate lags associated with biogeochemical and hydrologic N and P legacies in watersheds and rivers around the world (van Meter et al 2017, Chang et al 2021, Sarrazin et al 2021, Marques et al 2022, Zhou et al 2023). When calibrated, ELEMeNT has been capable of reproducing interannual variations in annual N yields, particularly in rivers that drain agricultural watersheds.

Equifinality is the idea that multiple model structures and/or parameter sets might be able to provide good model-data agreement for environmental systems (Beven 1993). The equifinality thesis rejects the idea that a single correct representation can necessarily be found, in favor of the idea that there might be many acceptable representations of an environmental system, and that this should be considered when assessing uncertainty (Beven 2006). This becomes important when models are used for purposes of mechanistic inference and not just curve fitting. Effective management decisions depend on an accurate understanding of the system of interest, which often depends on the accuracy of inferences made based on model results. Despite ELEMeNT’s success at mimicking real world data, concerns have been raised that equifinality may be an issue for the model’s usage, because parameters that represent N legacies, and therefore time lags, are poorly constrained by calibration against riverine data (Sarrazin et al 2021). In other words, wide ranges of parameter values may produce similarly good fits to data. This can obviously be problematic if a primary goal of modeling is to acquire mechanistic insight into system behavior.

An accurate understanding of delivery time lags is crucial for accurately predicting time scales associated with surface water clean-up. With models playing a crucial role in the application of watershed science, addressing equifinality in model structure and parameterization is important to furthering such understanding. The purpose of this study is to compare the accuracy and quality of several mechanistic models, containing different numbers and kinds of legacy N compartment representations (biogeochemical, hydrologic, or none) and governing parameters, at simulating annual riverine NO_3_–N yields. Inspired by ELEMeNT and other lagged mass balance-based work (e.g. Dupas et al 2020, Stackpoole et al 2021), we develop and calibrate six dynamic models for predicting annual NO_3_–N yield as a function of annual watershed net N surplus (briefly, summed inputs from atmospheric deposition, agricultural fertilizer, animal manure, crop and pasture N fixation, minus N uptake by crops and pasture grasses), wastewater N, and annual river discharge at 254 specific riverine sites across the continental US. These investigations are part of an effort to identify model construct(s) that accurately reproduce annual riverine N mass export for the ‘right’ reasons (i.e. that are physically plausible and internally consistent) for the purpose of gaining mechanistic insights into key processes operating at whole-watershed scales, and so that models can be used to confidently anticipate riverine N export under changing watershed N loading boundary conditions. Based on our results, we draw conclusions regarding more defensible general model structure(s) given observed NO_3_–N yield time series and discuss implications for N legacy stores and the role of lagged delivery in riverine N loading.

## 2. Data and methods

For data, we downloaded USGS-generated, annually summarized Weighted Regression on Time, Discharge, and Season (WRTDS)-modeled NO_3_–N yield time series data for 266 riverine sites (Murphy et al 2021) and their corresponding watershed boundary shapefiles (Falcone et al 2017) across the conterminous US. Watersheds for the sites ranged in area from ten to nearly three million square km^2^ and ranged widely in terms of dominant land uses/covers (supplemental information tables S1 and S2). To develop annual watershed net N surplus time series ($N_{\text{surp}}(t)$) for each site, we used the publicly-available ‘TREND-nitrogen’ dataset (Byrnes et al 2020a, 2020b), which includes estimates of various N sources and sinks, summarized annually by county, for the contiguous US for every year from 1930–2017 (Byrnes et al 2020b). We developed $N_{\text{surp}}(t)$ time series for each watershed using scripts written in the *R* programming language (version 4.2.1) that allocate N sources and sinks for each watershed based on watershed boundary-county polygon overlap, under the assumption that the TREND-nitrogen inputs are distributed homogeneously across each county. This assumption should entail minimal error in N input estimates for watersheds large enough to overlap multiple counties, though it seems likely to introduce errors for watersheds smaller than county scale. Although a full analysis of the impact of watershed areas was beyond the scope of this study, we note that the median watershed area for included sites was about 2000 km^2^, which is roughly comparable to the median area for counties in the conterminous US. We calculated $N_{\text{surp}}(t)$ for each year as the sum of annual N inputs from atmospheric deposition (NO_3_–N and NH_4_–N), agricultural fertilizer and manure (from cows, horses, sheep, chickens, pigs, and goats), and crop and pasture N fixation, minus N taken up by crops and pasture grasses. $N_{\text{surp}}(t)$ therefore represents the annual surplus of N remaining on the landscape following crop removal and, ignoring any concurrent (i.e. same year) losses to leaching or runoff.

We generated approximate point source N loads from human waste ($N_{\text{WWTP}}(t)$) for each watershed analogously, using data from the TREND-nitrogen ‘Human.csv’ file (Byrnes et al 2020b), which contains estimated N loads based on population census data. This file provides estimates not of direct point source N loading to surface waters, but of N in human waste generated within the watershed. Nonetheless, like Chang et al (2021), we assumed that it provides useful quantitative information on point source N loads. The available data do not distinguish between N discharged to sewerage and N discharged to septic systems or elsewhere. In our models the loads are treated as if they are delivered directly to surface water. That is, unlike $N_{\text{surp}}$, $N_{\text{WWTP}}$ loads are not assumed to pass through soil or groundwater. Losses are however assumed to occur at rates that are functions of discharge (supplemental information, text S1). In-stream removal and losses that occur during passage through treatment plants are implicitly lumped together in this empirical representation. Wastewater N as a portion of total N inputs (i.e. site-mean $N_{\text{WWTP}}/\left(N_{\text{surp}}(t)+N_{\text{WWTP}}(t)\right)$ ranged from 0.4% to 81.4% across the sites, with mean and median values of 11.8% and 5.3%, respectively.

Of the 266 sites, we eliminated any that had missing years in their continuous WRTDS records (ten sites). Because our models are mass-balanced based, we also eliminated two sites for which the long-term mean annual riverine N yield exceeded the corresponding mean annual N inputs from nonpoint and point sources (i.e. $N_{\text{surp}}(t)+N_{\text{WWTP}}(t)$, leaving a total of 254 sites. One of these sites, located in northern Vermont, has a watershed area less than 1% of the area of the county within which it resides. Its yield exceedance therefore seems likely to be a function of mismatch in spatial resolution with the county-scale TREND N source data. The other site is located on the Atchafalaya River, which branches off the Mississippi River in southern Louisiana. Although the actual drainage to this site logically includes all drainage to the Mississippi River up to the point where the two rivers diverge, the watershed boundary shapefile provided for this site includes only a small portion of the lower Mississippi River’s drainage (and none of the upper Midwestern corn belt, for example). Its yield exceedance therefore seems likely to be the result of severe underestimation of its area-normalized N surplus.

For each site we used the entire continuous time series of annual WRTDS output for model calibration. Specifically, we made use of the WRTDS ‘Flux_K’ records, which are estimates of annual riverine loads generated using the WRTDS-K model. This model improves upon the original WRTDS code by including a first-order autoregressive process to account for autocorrelation among model residuals (Zhang and Hirsch 2019). These time series ranged in length from 11 years (2007–2017) at seven sites, to 46 years (1972–2017) at 40 sites, with mean and median lengths of 27 and 26 years, respectively. The geographic distribution of sites encompasses much of the continental US but is weighted toward northeastern and lower Midwestern locations (figure 1). Based on the 2011 National Land Cover Dataset (NLCD) as indexed in StreamCat (Hill et al 2016), watersheds that drain to the 254 sites cover a range of land uses, from undeveloped to heavily agricultural and heavily urbanized (supplemental information, tables S1 and S2).

Like previous researchers (Schaefer et al 2009, Dupas et al 2015, Compton et al 2020), we found long term site-mean N yield to correlate with long term site-mean N inputs $\left(N_{\text{surp}}(t)+N_{\text{WWTP}}(t)\right)$ across the sites (figure 2). Site-mean fractional export as NO_3_–N (calculated on a site-by-site basis from watershed N inputs and WRTDS yields) ranged from 0.16% to 76.0% of total N inputs across the 254 sites, with average mean export across all sites of 11.4%. By comparison, Howarth et al (1996), Boyer et al (2002), Han and Allan (2008) and Swaney et al (2012) all reported mean TN export to be about 25% of watershed N inputs in various US, Chinese, and European rivers. Given that none of these researchers included N from animal manure or human waste among their inputs, and that they quantified TN export rather than NO_3_–N export, the smaller fractional yields in our study are not surprising.

The CV (coefficient of variation) of mean $N_{\text{surp}}(t)$ across sites is 59.2, whereas the CVs for $N_{\text{surp}}(t)$ across time at individual sites range from 3.2 to 60.6, with a mean of 12.5. Spatial variability in mean $N_{\text{surp}}(t)$ therefore exceeds temporal variability in annual $N_{\text{surp}}(t)$ at all but one of our sites. The evident relationship between mean N input and export seen in figure 2 and in the cited studies is evidence that riverine N yields are indeed influenced by watershed N inputs (and not just hydrology), consistent with findings from various studies (e.g. Howarth et al 1996, Boyer et al 2002, Han and Allan 2008, Capel et al 2018), and with the afore-mentioned paradigm 2.

Using the TREND-nitrogen-based annual $N_{\text{surp}}(t)$ and $N_{\text{WWTP}}(t)$ watershed time series as input, we developed scripts in R that implemented six models (M1–M6; table 1 and supporting information figures 1–6) and auto-calibrated each model against each of the 254 site NO_3_–N yield datasets. Each model generates as output separately calculated point source-derived and nonpoint source-derived N yields (N_out,PS_ and N_out,NPS_), which are then summed to compare with independent estimates of NO_3_–N yields (N_out_). Models M1 through M6 represent a progression from greater to lesser complexity, in that order. Accordingly, the six models vary in detail, with N passing through one to three chemical pools or compartments (soil organic nitrogen [SON pool], soil inorganic nitrogen [SIN pool], and groundwater inorganic nitrogen [GIN compartment]—see supplemental figures S1–S6), depending on the model, on its way to becoming exported riverine NO_3_–N (supporting information text S1). All models make use only of area-normalized annual riverine discharge (*v*), $N_{\text{surp}}(t)$, and $N_{\text{WWTP}}(t)$ as inputs. M1 is similar in structure to ELEMeNT, and the other five models are akin to M1 with various pieces removed.

In models that include an SON pool (i.e. biogeochemical legacy store, *M*_a_), we represented that pool as comprising accumulated N annual surpluses ($N_{\text{surp}}(t)$) minus a fraction lost to the SIN pool during each annual time step. Note that we chose to employ ‘*M*_a_’ and ‘*M*_s_’ to refer to the SON and SIN pools respectively, for consistency with ELEMeNT’s nomenclature (van Meter et al 2017). Potentially confusingly, both these N pools are described as being physically located in a ‘Source Zone’ in ELEMeNT, corresponding to the soil root zone. It is for this reason that we refer to these masses as ‘pools’ rather than compartments, unlike the groundwater within which GIN resides, which at least conceptually represents a real physical compartment. The *M*_a_ and *M*_s_ pools should not be confused with the M1–M6 designations we use to refer to the models in our study.

The N loss from SON to SIN occurs in a first-order manner governed by a mineralization coefficient, *k*_a_. In models M1 and M2, we modeled the GIN compartment as a completely mixed reactor having a stationary, exponential distribution of transit times with mean *τ*_g_, and subject to N loss (e.g. via denitrification) at rate *γ*. We modeled the SIN pool’s export flux in each model (referred to in supplemental information figures 1–6 as either ‘*M*_s_’ or ‘N_out,nps_’, depending on whether export from the pool represents flux to GIN or directly to riverine yield, respectively), as a completely mixed system with a first-order loss coefficient *λ*_s_, and with annually-updated transit times calculated based on a characteristic path length *L* and annually-varying area-normalized river discharge, *v*. For all six models, we represented point source-based N contributions to riverine yield using the same simple, empirically based formula. See supporting information (text S1) for a detailed explanation of the models and their development.

To compare model NO_3_–N yield predictions to WRTDS yield estimates (i.e. ‘observed data’), we used two summary measures: NSE (Nash-Sutcliffe Efficiency) and AICc (the corrected Akaike Information Criterion). Like a coefficient of determination (*R*^2^), the NSE statistic provides a measure of a time series model’s goodness of fit to the data in a summed-squared-error sense (Moriasi et al 2007), referred to here as model ‘accuracy’. Although there is no consensus on what constitutes a ‘good’ NSE value for watershed nutrient modeling conducted at annual time scales, for N and P modeling at monthly scales, Moriasi et al (2015) suggest that NSE > 0.65 be considered ‘very good’. For each model/site combination, our calibration process sought sets of parameter values that minimized summed squared deviations between observed data (WRTDS) and predicted NO_3_–N yield time series, and therefore optimized NSE scores and accuracy. However, because models with more parameters are more flexible, they tend to generate lower mean squared errors, which means that metrics like NSE cannot be used for choosing the best model among models with different numbers of parameters (James et al 2013). For that purpose, we employed AICc, which provides an indication of a model’s ‘quality’ relative to other models (Burnham and Anderson 2002, Andrade et al 2021). Unlike NSE, AICc incorporates a term whose magnitude increases with the number of model parameters to penalize overfitting. AICc is a version of the AIC statistic, modified to account for effects of small sample sizes, such as when the number of samples divided by the number of parameters is <40 (Symonds and Moussali 2011), as was the case for some of our model/time series combinations. Because the absolute magnitude of AICc, like AIC, has no real meaning, in comparisons between models, researchers often focus on ΔAICc (or ΔAIC), i.e. the difference between the lowest AICc and that produced by each individual model (Richards 2005). Models are generally considered to be of equal quality when ΔAICc <2, and to be of significantly different quality when ΔAICc > 10 (Burnham and Anderson 2002). In this work, we primarily use AICc as a ranking tool to identify the highest quality model at each site, without regard to whether differences between individual models are or are not significant according to the Burnham and Anderson criteria. However, in a test separate from the inter-model comparison, wherein we repeatedly recalibrated our most parsimonious model (M6) using $N_{\text{surp}}(t)$ input time series delayed in increments of from 1 to 20 years, we used distributions of ΔAICcs (referenced to the AICcs generated by non-lagged input) to empirically investigate the significance of model quality changes as a function of N input lag.

## 3. Results and discussion

Despite their differences in structure, all six calibrated models fit most of the datasets quite well, with median NSEs for simulated annual time series that ranged from 0.79 to 0.84 across models (figure 3). Figure 3(b) shows a general decrease in median AICc (i.e. an increase in model quality) from more complex to less complex models. The NSE values for all six models compare well with accuracies reported for ELEMeNT in representing other river systems (e.g. Chang et al 2021).

In tallying the 254 sites by best-performing model, we found the sites to be fairly well distributed across the models (table 3). NSE differences among models at a given site were often small, ranging from 0.004 to 1.09, with a median of 0.10. However, in terms of AICc, M6 outperformed all other models at 60% (153) of sites. Model M5 (the other single-compartment model, though with a total of four parameters instead of three) was the next-best performer, generating the lowest AICcs at 22% (55) of sites (table 3). At over 86% of sites, models with either three or four parameters (M4, M5, and M6) demonstrated superior quality compared to models with five to seven parameters (M1, M2, and M3). Figure 4 shows the geographical distribution of the 254 sites in terms of highest accuracy (based on NSE) and highest quality (based on AICc) model. Sites are well distributed geographically according to both metrics with little obvious spatial clustering, except perhaps for model M1, which had higher NSEs than the other models at a set of sites located along and near the Gulf coasts of Florida, Georgia, and Alabama.

We chose six examples to illustrate cases where models M1–M6 performed best (figure 5). Despite large differences in the watershed attributes (area, geographic location, land use/land cover) at each of the sites and relative contribution to total N input from point source-based N (table 2), all six models accurately replicated overall trends and, perhaps as importantly, interannual fluctuations in observed NO_3_–N yield at each site. Each subfigure represents a case where one of the models (M1–M6) outperformed all the others in terms of AICc (though not necessarily in terms of NSE), as indicated in the subfigure annotations. The outputs of all six models are superimposed on top of each other in gray (or black in the case of the model with the lowest AICc) on each subfigure, along with the observed data shown in red. This was the case not just in the six examples shown in figure 5, but for most of the 254 modeled sites (supplemental information figure 10).

Although their accuracies (NSE) were usually slightly higher, models with more pools/compartments and parameters tended to exhibit poorer quality (AICc). In other words, inclusion of pools/compartments that represent specific legacy stores (SON and/or GIN) tended to increase model accuracy, but not by enough to outweigh the decrease in model quality brought about by inclusion of the associated extra parameters. Even at sites where models that included such features outperformed simpler models in terms of AICc however, the calibrated magnitudes of parameters associated with these compartments imply that lagged contributions to N loading are negligible. The phenomenon is evident in the examples shown in figures 6 and 7, wherein large values of rate coefficients (*k*_a_ and *γ*, respectively) essentially negate the presence, in the simulations, of substantial biogeochemical and hydrologic legacy N stores respectively, along with associated lagged N contributions to riverine N flux.

Median AICc increased significantly with the number of model parameters (figure 8), and model M6, with just three parameters in total, generated the lowest median AICc (figure 3(b)). The calibration process searched multidimensional parameter space(s) for sets of values associated with the global minimum summed squared model errors. This process often selected sets of parameters governing the legacy compartments that rendered these compartments little more than pass-through entities. Thus, irrespective of which model exhibited the highest quality, lagged delivery as a meaningful driver of annual riverine N loading at the sites evaluated here was essentially absent. It is worth noting that an Akaike-weighted multi-model average (Kolassa 2011) of the M1–M6 outputs generated higher NSEs than did M6 by itself, at the vast majority of sites (199).

At 230 sites, the optimized mineralization coefficients (*k*_a_) of one or more of the models that included an SON pool (M1, M3, and M5) were sufficiently high that the flux of mineralized N to the SIN pool (*k*_a_*M_a_) was indistinguishable from $N_{\text{surp}}(t)$ itself, indicating that N accumulation in the SON pool (i.e. biogeochemical legacy) was essentially non-existent (figures 6(b) and (c)), meaning that biogeochemical legacy can be ignored. At 72 sites, this was the case for all three models having SON compartments (e.g. figure 6(a)). Similarly, optimized mean hydrologic transit times and NO_3_–N loss coefficients for the GIN compartment (*τ*_g_ and *γ*, respectively) of the models M1 and M2 were, at most sites, small enough and large enough, respectively, that almost all the NO_3_–N delivered to surface water was derived from same-year NO_3_–N flux from the GIN compartment. This indicates essentially no lag in delivery or substantial N mass languishing in the GIN compartment, meaning that hydrologic legacy can also be ignored. In a few examples mean transit times were up to decades in length, however the associated high subsurface loss (i.e. denitrification) rates essentially negated all but same-year transport of NO_3_–N from the SIN pool to surface water via the GIN compartment (figure 7). Considered only in isolation, such long hydrologic transit times could lead to a misimpression that similarly long delivery lag times for NO_3_–N are operative. For this reason, to properly infer physical meaning from calibrated multiparameter models, we suggest that calibrated parameters should be considered together as sets (e.g. mean transit times and loss rates), rather than as isolated entities.

As mentioned, an aspect of our analysis involved recalibrating our highest quality (and most parsimonious) model (M6), using $N_{\text{surp}}(t)$ time series that were intentionally delayed by increments of from 1 to 20 years. In doing so, we were motivated by our observation that interannual variations in $N_{\text{surp}}(t)$ tend to be much milder than interannual variations in discharge, and that the influence of $N_{\text{surp}}(t)$ on interannual NO_3_–N yield fluctuations may therefore be challenging to discern. We thus sought to interrogate, from a different perspective, our finding that including legacy compartments did not increase model quality and the associated implication that lagged delivery does not detectably influence riverine NO_3_–N loads. Because there is no plausible mechanism by which point source-based N might in general be delayed for >1 year before discharge to rivers, we left the $N_{\text{WWTP}}(t)$ inputs unchanged in these recalibrations. In this test we found that median model accuracy declined monotonically, though gradually, with increasing input lag (figure 9(a)). However, the corresponding declines in median model quality (figure 9(b)) were sufficiently gradual that models with lags up to 3 years show no improvement over zero lag models, and models with lags longer than ten years are not supported, based upon the Burnham and Anderson (2002) criteria. Despite this overall trend, it is important to note that model quality for a few sites did improve significantly with lags of various magnitudes, even lags for which median ΔAICcs were not significant (i.e. <10). Cumulative ΔAICc density functions (CDFs) for each lag are shown in the supplemental information (figure S7). The gradual changes in median NSE and AICc with increasing lag (figure 9) are likely a consequence of the fact that the $N_{\text{surp}}(t)$ inputs themselves displayed substantial autocorrelation over multi-year time scales, as demonstrated by stronger linear relationships between time series offset by lags of from one to ten years (supporting information figures S8–S9). Based on this result, it appears that the influence of nonpoint source inputs on riverine N lagged by up to about ten years may be difficult to distinguish from contributions originating from same-year $N_{\text{surp}}(t)$.

The surprising success of M6 at accurately simulating annual riverine NO_3_–N yields demonstrates that entire watershed/river systems can be represented as if they were CSTRs (Continuously Stirred Tank Reactors) operating in punctuated, temporary steady-state, wherein residence time is an annual-average construct that updates as annual discharge varies from year to year. In this conceptualization, the quotient *λ*_s_*L*/*v* is a year-specific whole-watershed Damköhler number (Chapra 1997), which varies inversely with annual v (area-normalized discharge). All physical processes, including overland runoff and subsurface transport, which translate $N_{\text{surp}}(t)$ into riverine NO_3_–N(*t*) within a year’s time span, are represented in aggregate by the model’s sole parameter, *λ*_s_*L*, as are all biogeochemical processes, including metabolism of reduced N forms (e.g. SON) and losses via denitrification. Examining the form of the model (supplemental information equation (S27)), we note that when the Damköhler number is substantially greater than 1 (which was the case in most years at nearly all sites), the model simplifies to approximate proportionality between NO_3_–N load and the product of discharge and $N_{\text{surp}}(t)$. Our results thus seem to generally support the second paradigm discussed in the Introduction and fail to strongly support the existence of influential watershed legacy N stores or greater-than-annual delivery lags at the sites we evaluated.

The high performance of all six models at matching the various datasets despite representing N fate and transport in fundamentally different ways illustrates that substantial equifinality may be at play in mechanistic representations of watershed N behavior. That is, one may arrive at the ‘right’ answer for the ‘wrong’ reason. This again is less of an issue when models are used for representation than when they are used for gaining mechanistic insight. Because we approached the question of how best to model annual NO_3_–N yields as functions of N inputs and riverine discharge mechanistically, we treated nonpoint source inputs ($N_{\text{surp}}(t)$) and point source inputs ($N_{\text{WWTP}}(t)$) separately, with each processed by a separate computational mechanism governed by its own set of parameters. This is why M6 is a three-parameter, rather than a one-parameter model. Despite its parsimony relative to the other five models, there is therefore an element of parameter equifinality present, even in M6. The equation we chose to model contributions to riverine yields from point source-based yields is similar to how M6 represents contributions from nonpoint source-based yields (see equations (S31) and (S27), respectively, in the supplemental information). For many sites, the calibrated parameter sets included ‘*B*’ values (supplemental text S1) optimized at −1. In such cases, riverine yield is represented in M6 as essentially the summed output of two separate CSTRs representing the processing of point source and nonpoint source inputs, respectively. This raises the possibility that an even simpler, single-parameter model, with the $N_{\text{surp}}(t)$ and $N_{\text{WWTP}}(t)$ inputs combined into one aggregate time series, might suffice to represent riverine yields as a function of total same-year N watershed inputs.

Both the input and output datasets used in our analyses involve inevitable error and uncertainty. The input data, for example, are based on $N_{\text{surp}}(t)$ values interpolated to provide continual estimates over years lacking data and summarized at the county scale before being allocated to the watersheds of interest via simple spatial overlap. The output data similarly are annual NO_3_–N yield estimates generated by the WRTDS model (and published by the USGS) but treated here as ground truth data for purposes of model calibration. Any systematic bias in either of these data sources would influence model calibration and associated comparisons. Despite these uncertainties, the overall trend of parsimonious models outperforming more detailed ones is clear, across the 254 sites considered in this study.

## 4. Conclusions

The six models considered in this study represent watershed nonpoint source-based N chemical transformations, transport, and attenuation using contrasting levels of complexity and kinds of mechanistic representation. Despite their diversity, all models were able to match most of the 254 time series datasets quite well, generating NSE scores that indicate close agreement between models and data. This is a striking result given that some models included compartments representing legacy stores and some did not. Perhaps as important, for models that included legacy compartments, calibration via minimization of summed squared model errors usually selected values for governing parameters that rendered the magnitudes of these N stores negligible. Lower (better) AICcs were associated with models having fewer parameters, and the simplest model (M6), which included no legacy pools or compartments, outperformed the other five in terms of AICc at most sites. All of this would seem to negate the existence of legacy N stores and delivery lags, but caution in drawing such a conclusion is warranted. Fluctuations in annual discharge are clearly the primary driver of fluctuations in annual riverine N loading, but it is evident that annual watershed N inputs also influence annual N loads. The M6 model is nearly identical mathematically to the empirically based ‘paradigm 2’, according to which loads are proportional to the product of discharge and N surplus. However, annual N surpluses are themselves strongly autocorrelated, with mild interannual variations seemingly masking the ability to observe their influence on riverine loads. Recalibrations of M6 using incrementally lagged $N_{\text{surp}}(t)$ time series illustrate that the temporal connection between watershed N inputs and riverine N loads is fuzzy, with lags of up to about a decade generating AICcs that are, in the median, indistinguishable, according to the Burnham and Anderson (2002) criteria, from those generated using no lag. Although M6 was found to have the highest ‘quality’ among the models tested, and more parsimonious models generally outperformed more detailed ones, the fact that a multi-model average outperformed all six models (in terms of NSE) demonstrates that none of these models should be assumed to constitute a ‘correct’, or even best possible representation of underlying physical reality. Rather than truly mechanistic representation, models like M6 might better be thought of as providing a pseudo-mechanistic framework for empirically representing annual emergent behavior, at whole-watershed scales, of physical, chemical, and biological processes that are infinitely detailed, varied, and incompletely knowable at finer spatial and temporal scales where field measurements occur (Beven 2001). Future investigations will explore other model formulations and approaches to representing watershed and riverine transport of nutrients, including other forms of N, and possibly P as well.

## Supplementary Material

Supplement1

Supplementary material for this article is available online

## Figures and Tables

**Figure 1.**
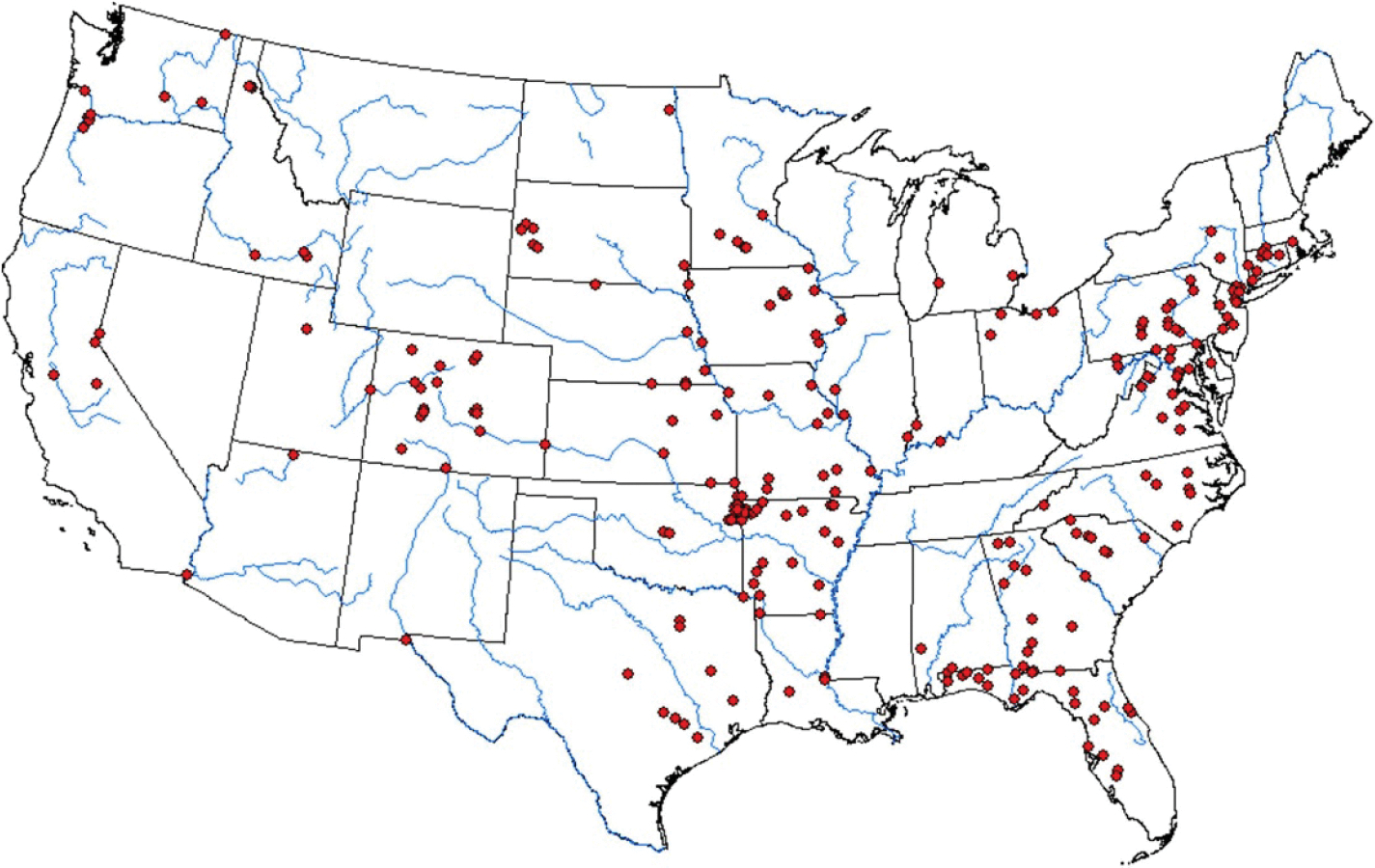
Locations of 254 USGS riverine NO_3_–N monitoring/WRTDS modeling sites evaluated in this study. See supplemental table S1 for watershed property summary.

**Figure 2.**
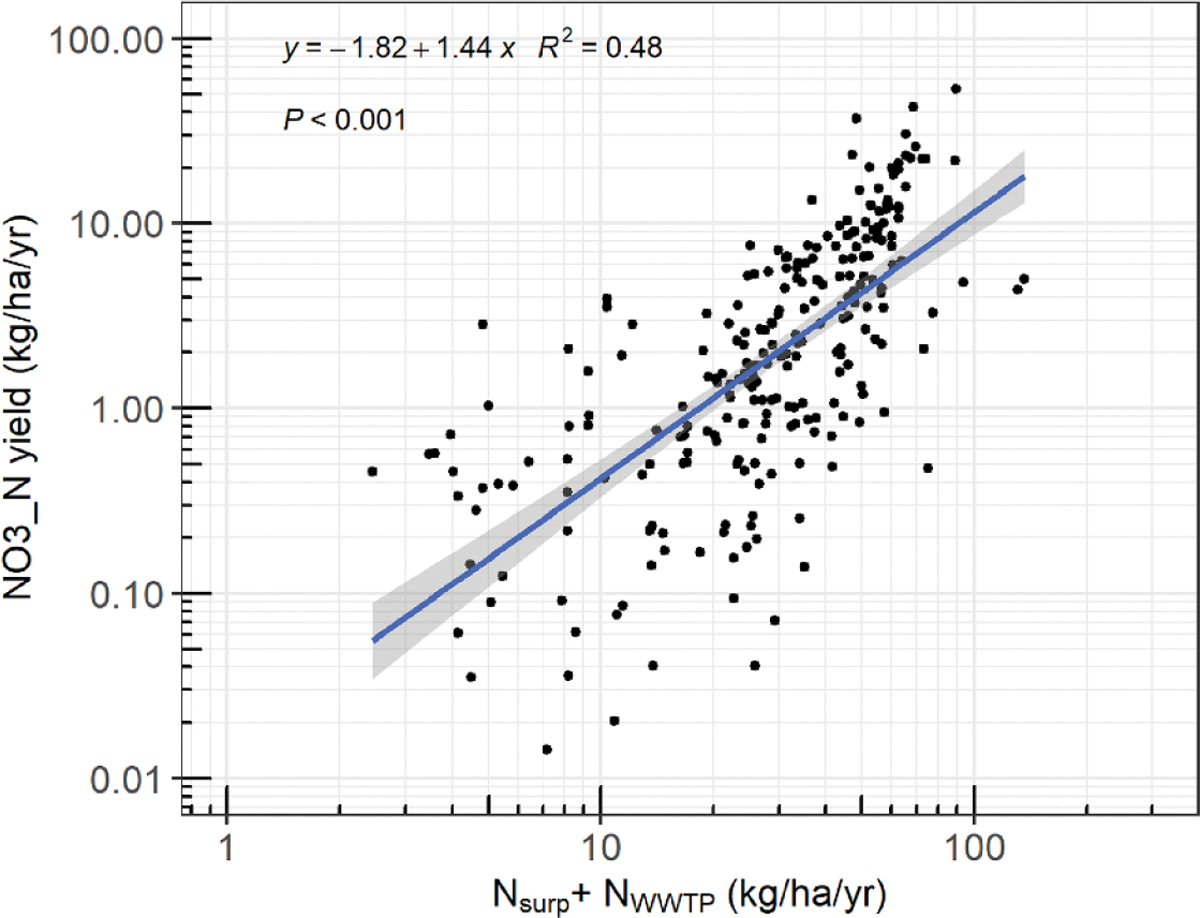
Mean NO_3_–N yield vs mean watershed total N inputs ($N_{\text{surp}}(t)+N_{\text{WWTP}}(t)$) at 254 riverine sites. Averages are calculated across all available years (range = 11–46 years) for both variables at each site. Regression line is shown with 95% confidence interval.

**Figure 3.**
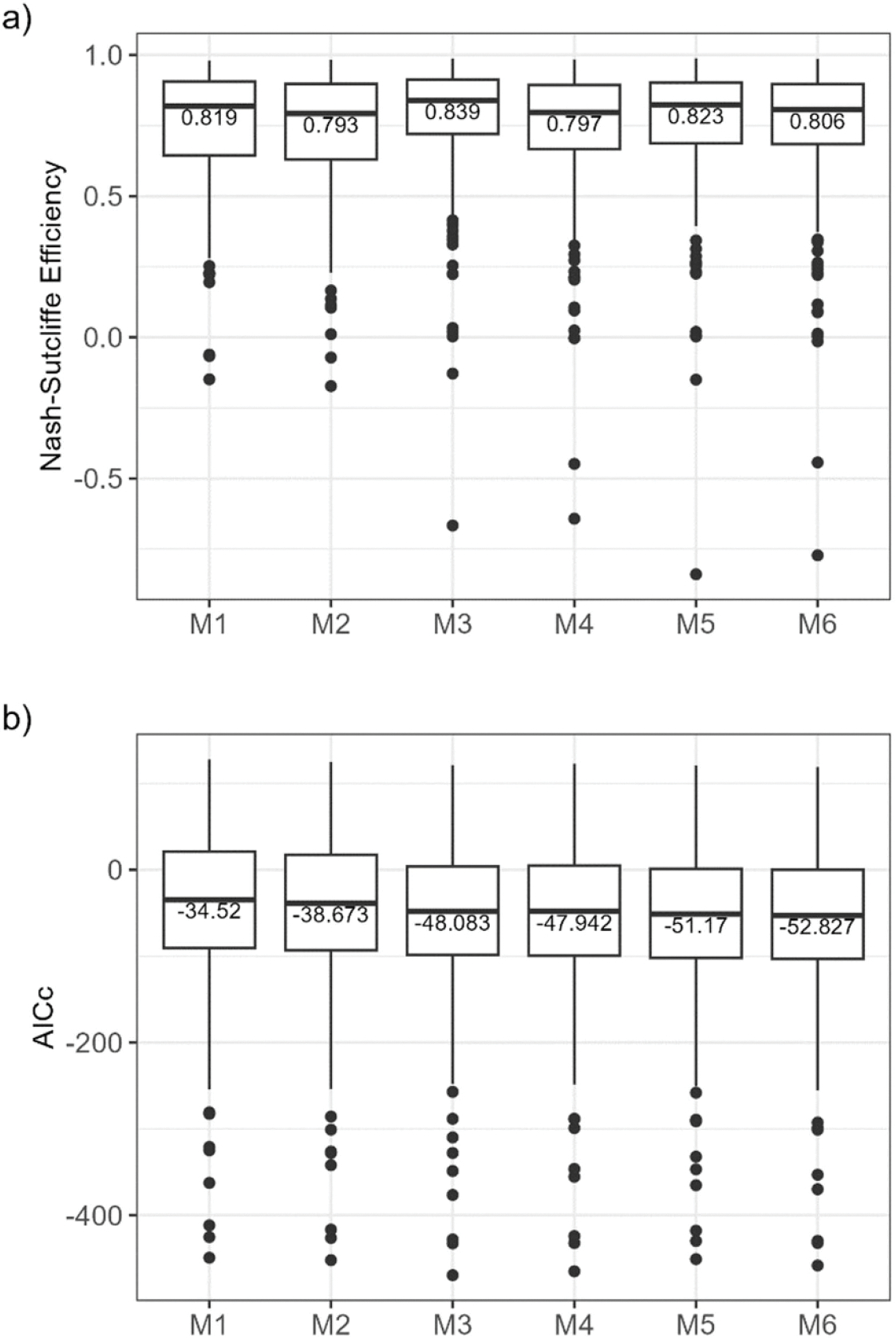
(a) Nash–Sutcliffe efficiencies (NSE) and (b) adjusted Akaike information criterion scores (AICc) for six models calibrated against each of 254 riverine annual NO_3_–N yield data sets. Median NSE and AICc values for each model are indicated on the figure.

**Figure 4.**
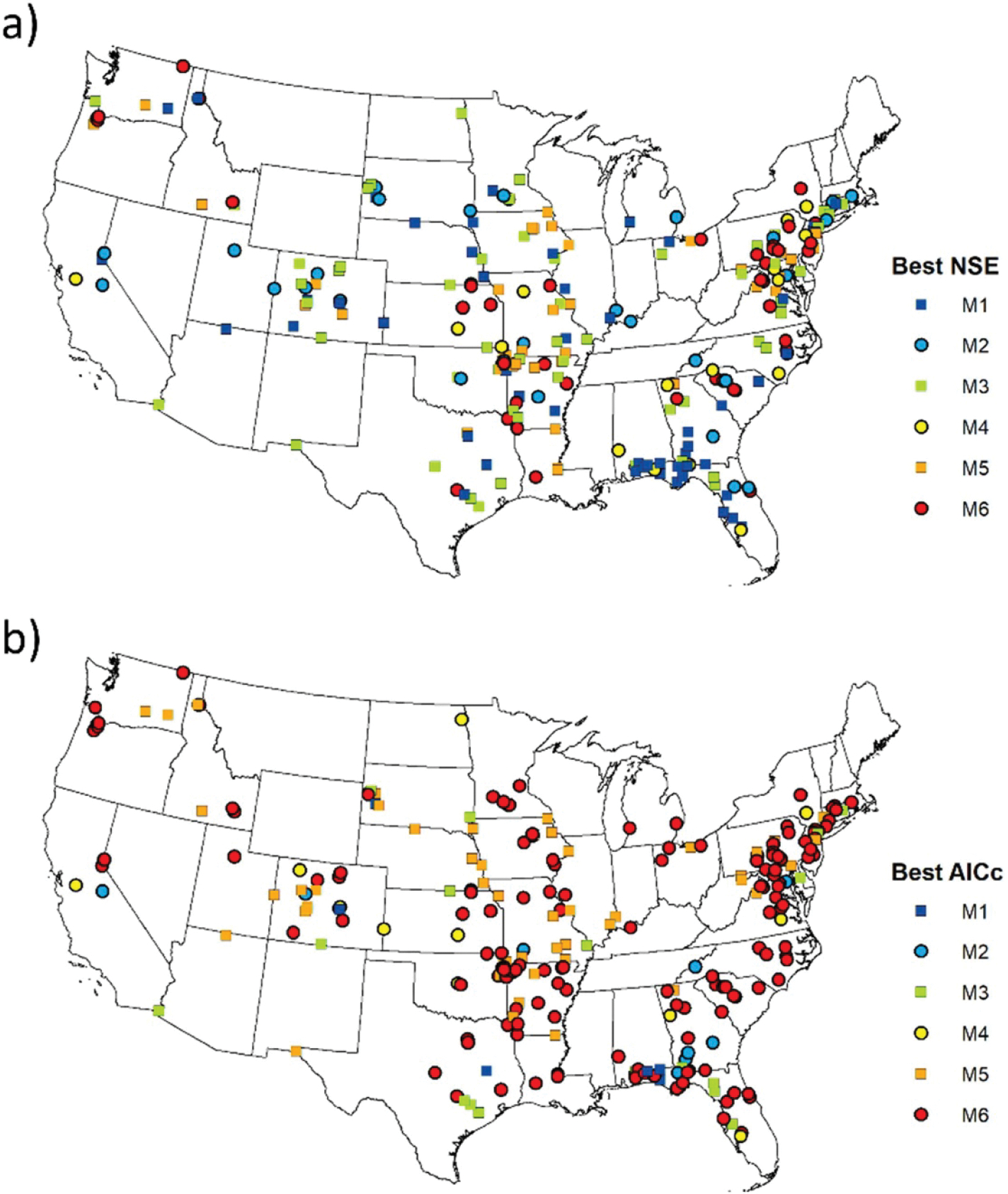
Modeled riverine NO_3_–N yield sites, color-coded by (a) most accurate model, based on NSE and (b) highest quality model, based on AICc.

**Figure 5.**
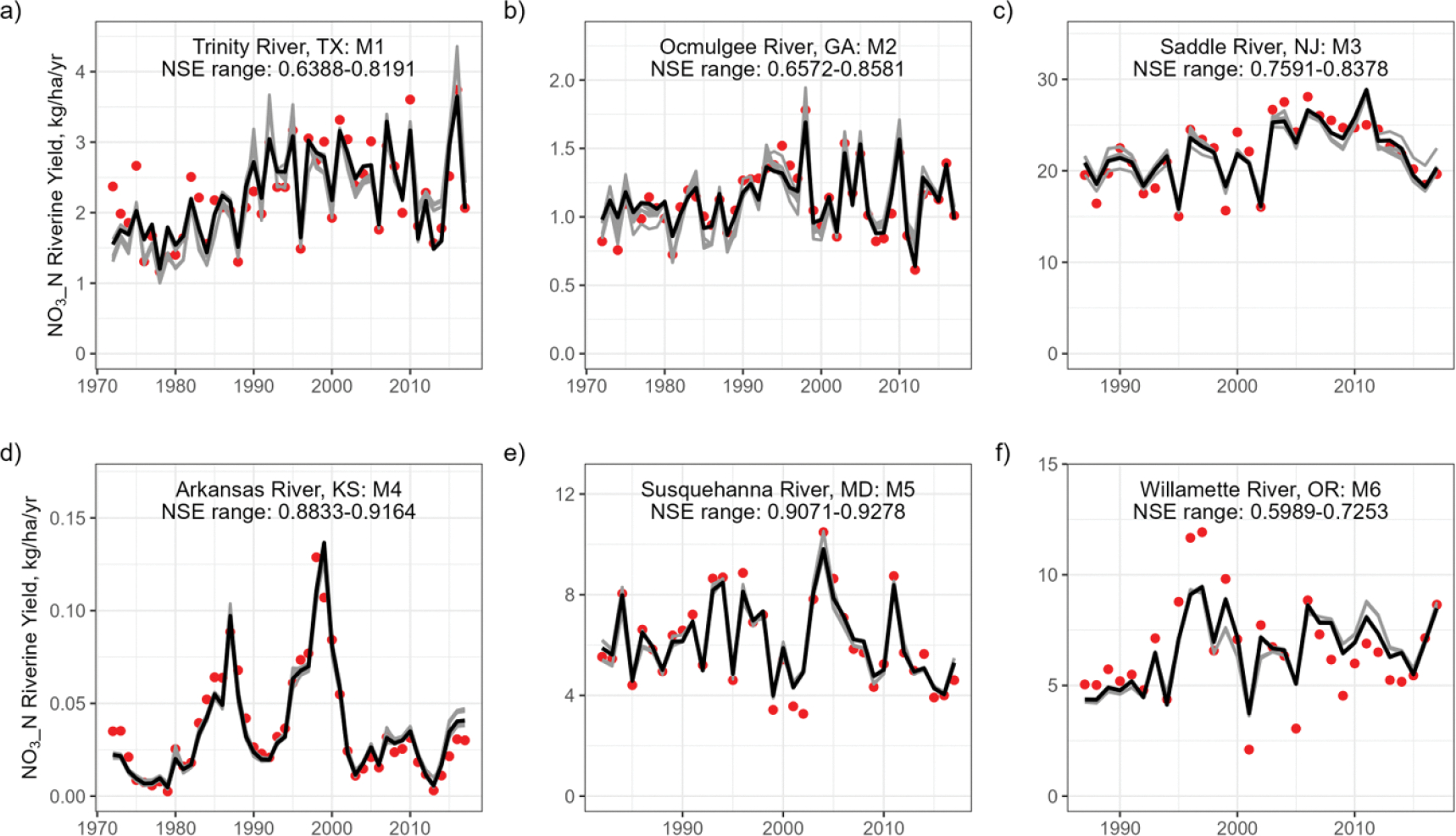
Time series plots showing results from the best performing model (M1–M6) at six example sites based on AICcs: (a) M1 at the Trinity River, TX (SITEID #8065350); (b) M2 at the Ocmulgee River, GA (SITEID #2215500); (c) M3 at the Saddle River, NJ (SITEID #1391500); (d) M4 at the Arkansas River, KS (SITEID #07137500_21247638); (e) M5 at the Susquehanna River, MD (SITEID #1578310); and (f) M6 at the Willamette River, OR (SITEID #14211720). Black lines represent NO_3_–N yields generated by the best-performing models at each site, gray lines represent NO_3_–N yields generated by the other six models, and red dots are observed data (WRTDS modeled).

**Figure 6.**
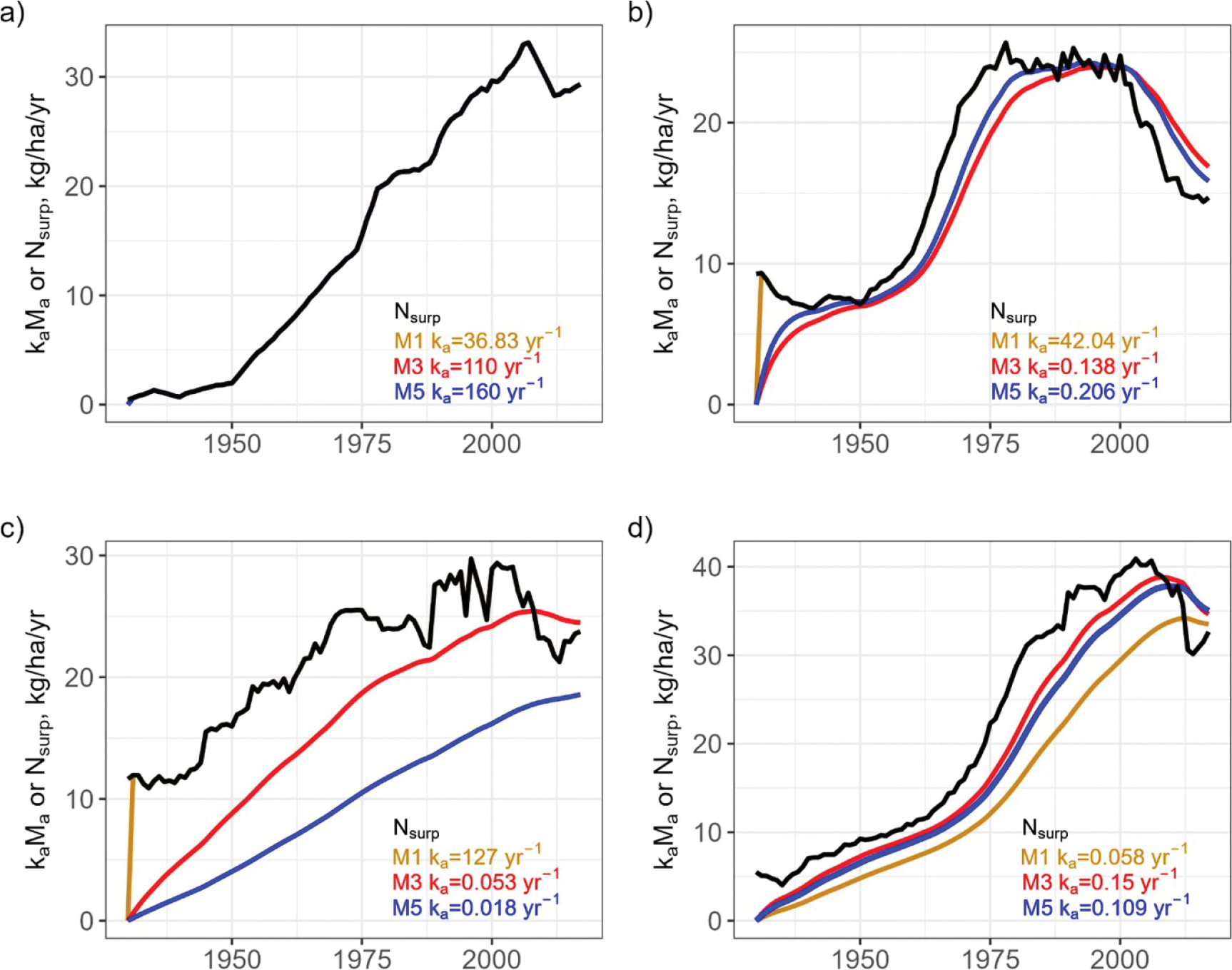
Watershed annual net N surplus ($N_{\text{surp}}(t)$) and calibrated N flux from SON zone (*k*_a_*M*_a_) at four example sites: (a) Willamette River, OR (SITEID #14211720); (b) Ocmulgee River, GA (SITEID #2215500); (c) Saddle River, NJ (SITEID #1391500); (d) Kings River, AR (site #arWHI0009A_ARDE). In subfigure (a), *k*_a_ values are sufficiently large that the N fluxes from models M1, M3 and M5 are all indistinguishable from $N_{\text{surp}}(t)$, thus their traces are essentially identical to and visually obscured by the black $N_{\text{surp}}(t)$ trace.

**Figure 7.**
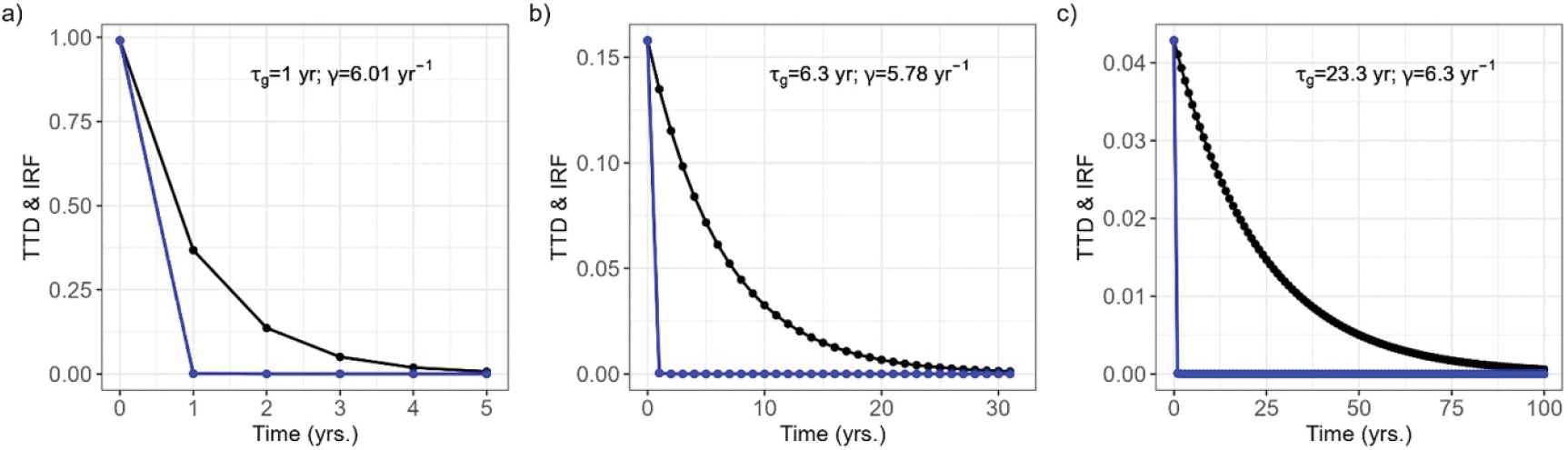
Groundwater transit time distributions (TTDs—black) and impulse response functions (IRFs—blue) for: (a) M1: Farmington River, CT (site #118995); (b) M2: Flint River, GA (site #2349605); (c) M2: Red River, AR (site #arRED0046). Mean GIN transit times (*τ*_g_) and NO_3_–N decay coefficients (*γ*) indicated on figures. These examples illustrate that even when mean groundwater transit times are as long as decades (black lines), associated loss rates of N (e.g. to denitrification) may effectively limit N transport to surface waters (blue lines) to same-year delivery.

**Figure 8.**
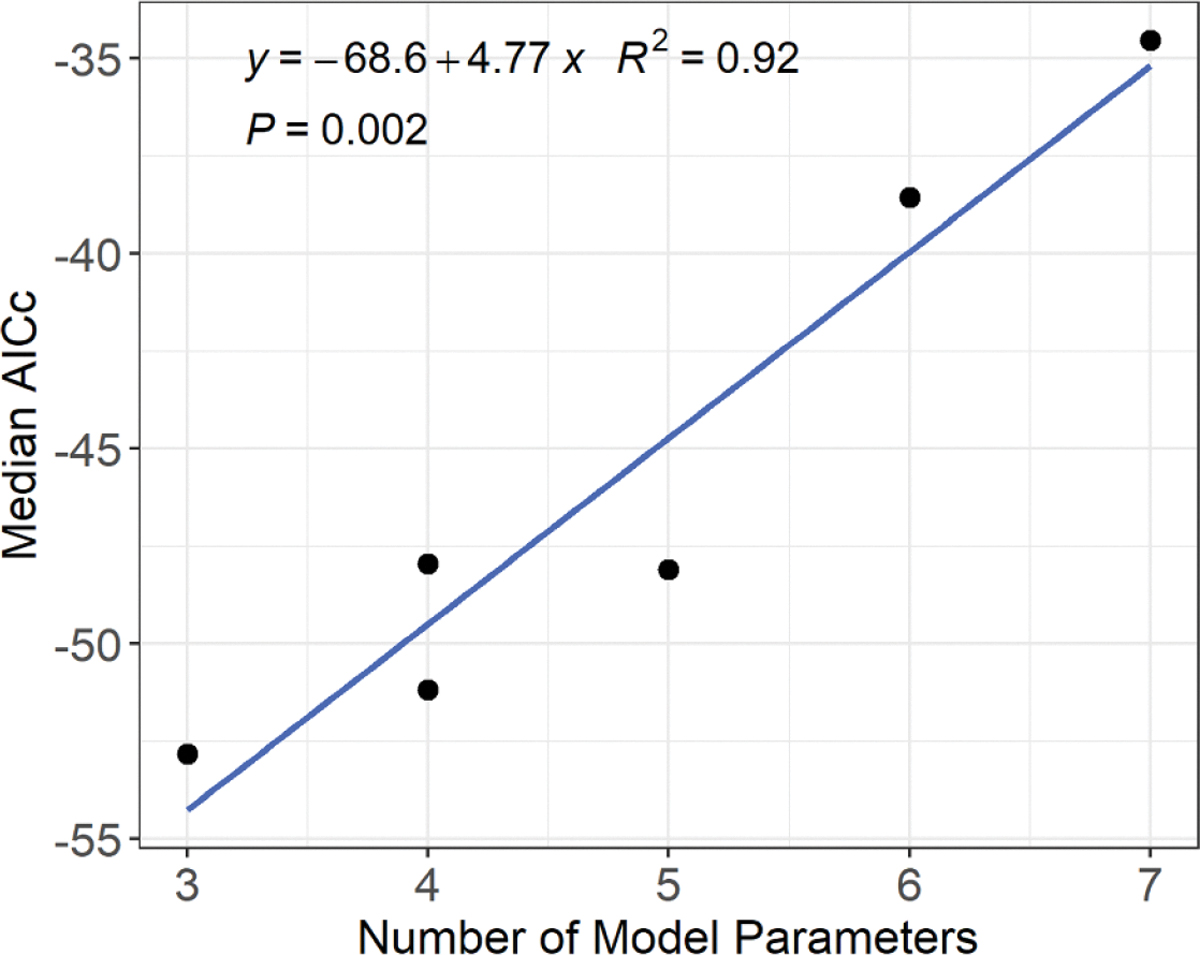
Median AICc vs number of model parameters.

**Figure 9.**
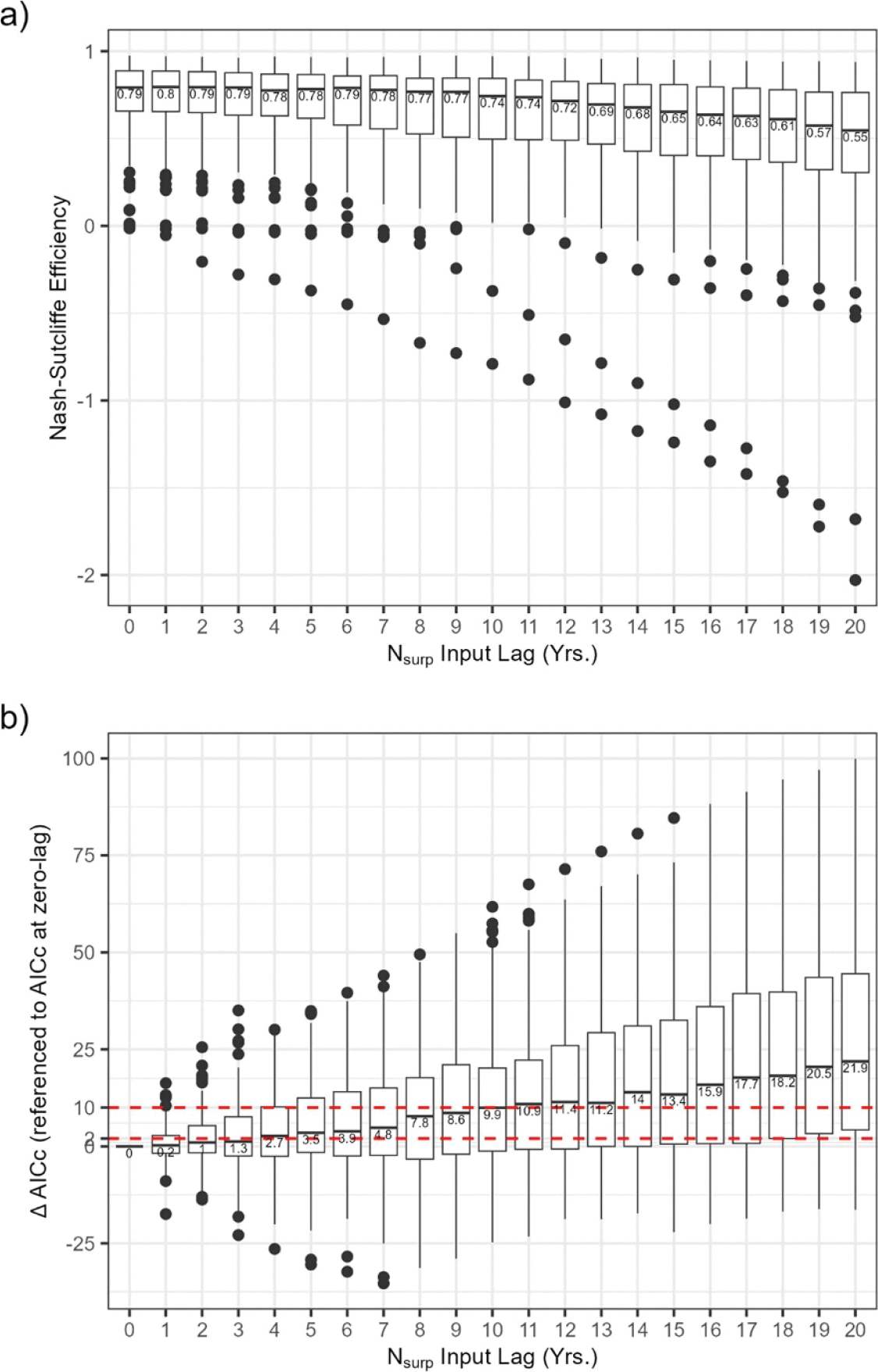
Boxplots of the performance metrics (a) NSEs and (b) ΔAICcs adjusted to site-specific minima of zero, for the M6 model calibrated using input $N_{\text{surp}}(t)$ lagged by 0–20 years, at the 144 sites with more than 20 years of WRTDS data. ΔAICc was calculated by adjusting the AICc score for each site by subtracting the AICc obtained at an input lag of zero years (i.e. no lag). Boxes in the figure indicate the interquartile range (25th to 75th percentiles). Upper and lower whiskers indicate the largest values that are no more than 1.5 times the interquartile range above the 75th percentile, and below the 25th percentile values, respectively. Individual dots are outlying points. Dashed red horizontal lines at 2 and 10 indicate levels associated with empirical support for significant differences, demarcating ΔAICc at values below and above which models can be considered of equal and significantly different quality, respectively (Burnham and Anderson 2002, p 70). Full cumulative ΔAIC distributions at each lag are shown in the supplemental information (figure S7).

**Table 1.** Six watershed NO_3_–N yield models (M1–M6) evaluated in this study (figures S1–S6 and text S1).

Model	Pools/compartments	Legacy/lag potentially represented in pools/compartments	Number of parameters^a^	Summary description
M1	SON, SIN, GIN	SON, SIN, GIN	7	Pools/compartment arranged in series. All prior years’ N_surp_ values potentially influence current year yields, through both biogeochemical and hydrologic legacies, as well as potentially through unspecified lags occurring in the SIN.
M2	SIN, GIN	SIN, GIN	6	Pool & compartment arranged in series. All prior years’ N_surp_ values potentially influence current year yields, through hydrologic legacy and/or SIN lags.
M3	SON, SIN	SON, SIN	5	Pools arranged in series. All prior years’ N_surp_ values potentially influence current year yields, through biogeochemical legacy and SIN lags.
M4	SIN	SIN	4	All prior years’ N_surp_ values potentially influence current year yields, through unspecified SIN lags only.
M5	SON, SIN	SON, SIN	4	Pools arranged in series. SIN is in punctuated steady-state. All prior years’ N_surp_ values potentially influence current year yields, through biogeochemical legacy only.
M6	SIN	NA	3	SIN is in punctuated steady-state. Only current year N_surp_ values influence current year yields.

a Equations associated with each model are provided in supporting information (text S1 and supplemental figures 1–6).

**Table 2.** Watershed properties of six example sites highlighted in figure 5.

Site	Watershed area (km^2^)	% Imperviousness	Total % urban^a^	Total % agriculture^a^	Total % forest^a^	WWTP % of Total N input^b^	Highest quality model^c^

Arkansas River, KS	63 277.6	0.7	1.1	6.9	14.9	6.8	M4
Ocmulgee River, GA	13 572.3	5.2	9.7	17.6	44.0	20.9	M2
Saddle River, NJ	157.9	25.0	43.3	0.6	12.4	71.0	M3
Susquehanna River, MD	70 160.7	2.2	4.1	24.3	61.3	8.0	M5
Trinity River, TX	35 961.7	7.2	12.3	27.5	14.0	19.1	M1
Willamette River, OR	28 921.7	3.2	5.5	21.1	54.9	9.1	M6

a From NLCD 2011, via StreamCat (Hill et al 2016).

b Mean $N_{\text{WWTP}}(t)/(N_{\text{surp}}(t)+N_{\text{WWTP}}(t)$ calculated based on information in TREND-nitrogen input files. Note that the WWTP estimates are based on human waste loading rates in each watershed.

c As determined based on AICc value.

**Table 3.** Best-performing NO_3_–N yield models at 254 riverine sites, in terms of both model accuracy (NSE) and model quality (AICc).

Model	Highest NSE site count	Lowest AICc site count

M1	58 (22.8%)	7 (2.8%)
M2	34 (13.4%)	10 (3.9%)
M3	61 (24.0%)	17 (6.7%)
M4	19 (7.5%)	12 (4.7%)
M5	40 (15.7%)	55 (21.7%)
M6	42 (16.5%)	153 (60.2%)

Values indicate the number of sites in each best-model category.

## Data Availability

The data that support the findings of this study are openly available at the following URL/DOI: https://data.usgs.gov/datacatalog/data/USGS:5fff2c75d34e592d8671ecfd, https://doi.pangaea.de/10.1594/PANGAEA.917583. Supplemental Information available at https://doi.org/10.1088/3033-4942/ae09d6/data1.
