# Supplementary material for: Accounting for legacy nitrogen stores does not improve the accuracy of riverine nitrate load model predictions
Source: Environ Res Water. Author manuscript; Available in PMC 2026 Oct 10. (PMC12828579; doi:10.1088/3033-4942/ae09d6)
Supplement: Supplementary file 1 [file NIHMS2117752-supplement-Supplement1.docx]

Supplemental Information for

**Accounting for legacy nitrogen stores does not improve the accuracy of riverine nitrate load model predictions**

James N. Carleton^1^ and Robert D. Sabo^1^

Office of Research and Development, Center for Public Health and Environmental Assessment, U.S. Environmental Protection Agency (Mail Code 8623R), 1200 Pennsylvania Ave NW, Washington, DC 20460, USA

**Contents of this file**

Text S1

Figures S1-S11

Table S1

Table S2

**Text S1. Model Development and Calibration**

*Nonpoint Source-Based Yields*

Based on published descriptions (Chang et al., 2021; Liu et al., 2021; Marques et al., 2022; van Meter et al., 2017), the ELEMeNT model treats watersheds as comprising land use specific units (one each for cropland, pastureland, and “other”), each of which contains a “source zone” corresponding to the soil root zone. Within these source zones, SON (soil organic nitrogen) is assumed to exist in two separate pools: one “active” (relatively labile), and one “protected” (relatively recalcitrant). These change over time because of annual inputs of fertilizer and other N sources to the landscape, and losses due to crop removal and mineralization. Mineralization of both SON pools (each occurring at its own rate, with the active pool’s being much faster) contributes mineral N (i.e., nitrate) to an inorganic N pool, somewhat vaguely also conceived as being located within the source, or root zone, and from where N leaches to a groundwater zone. The groundwater is assumed to be characterized by an exponential distribution of transit times to surface water, and thus in effect to act as a completely mixed compartment. Within the groundwater compartment N is subject to first order loss (i.e., via denitrification), and to loss via discharge to surface water. The N transported through the groundwater compartment constitutes the entirety of the nonpoint source contribution to riverine N yield, which is calculated on an annual time step.

Annual mass balance on the active SON pool is represented in the ELEMeNT model as:

$\frac{dM_{a}}{dt}=\left( 1-h \right)*N_{surp}(t)-k_{a}M_{a}$	(S1)

where M_a_ is the active-pool SON stock (kg/ha), N_surp_(t) is annual net N input flux to the landscape (kg ha^-1^ yr^-1^) (similar to those calculated in Byrnes et al., 2020, Sabo et al., 2019, and Sabo et al., 2021), k_a_ is active SON mineralization rate (yr^-1^), and h is a fractional “protection coefficient” (dimensionless) that partitions N_surp_(t) between the protected and active pools. Mass balance on the protected pool SON is, similarly,

$\frac{dM_{p}}{dt}=h*N_{surp}(t)-k_{p}M_{p}$	(S2)

where M_p_ and k_p_ are protected pool SON and its mineralization rate, respectively. Active and protected SON pools both feed mineralized N into a common soil inorganic nitrogen pool, M_s_ (kg/ha), whose mass balance is given by

$\frac{dM_{s}}{dt}=k_{a}M_{a}+k_{p}M_{p}-\lambda_{s}M_{s}-J_{s}$	(S3)

where λ_s_ is a denitrification/loss rate (yr^-1^) and J_s_ is a “source function” representing N flux (kg ha^-1^ yr^-1^) from the soil inorganic N pool to groundwater. Published protected-pool SON mineralization rates are orders of magnitude smaller than corresponding active-pool SON mineralization rates (e.g., van Meter et al 2017a, Table S2.5), suggesting that the relative contribution to source N from protected-pool SON is negligible. Assuming that separate active and protected SON pools actually exist, it is conceivable that land use changes could disrupt recalcitrant SON in such a manner as to convert it into labile SON, increasing the size of that pool. However, neither ELEMeNT nor any of our models include a means of representing such a mechanism.

*Nonpoint Source-Based Yields: Models M1-M6*

Focusing on the active SON pool, we define a mass flux term from that pool to the inorganic N pool, which for clarity we refer to as the “SIN” (Soil Organic N) pool, as

$J_{m}=k_{a}M_{a}$	(S4)

which allows S1 to be re-expressed as

$\frac{dJ_{m}}{dt}=\left( 1-h \right)*k_{a}N_{surp}\left( t \right)-J_{m}$	(S5)

S5 is an ordinary differential equation whose general solution is

$J_{m}(t)=c_{0}e^{-k_{a}t}+\left( 1-h \right)*N_{surp}\left( t \right)$	(S6)

where c_0_ is a constant that in this case is defined in terms of the mass flux at the beginning of a time step:

$c_{0}=J_{m}\left( 0 \right)-\left( 1-h \right)*N_{surp}(t)$	(S7)

When k_a_ is sufficiently large, the exponential term in eq. S6 becomes negligible, and mass flux at the end of each time step is then approximately proportional to that time step’s N_surp_(t):

$J_{m}(t)\cong\left( 1-h \right)*N_{surp}(t)$	(S8)

The definition of negligible here is admittedly ambiguous. If we assume for the sake of argument that it means 5% or less of the total flux, then a value of ~3 yr^-1^ or greater for k_a_ would suffice to justify the S8 approximation if h is also small. The physical implication is that N does not appreciably “build up” from year to year in the form of SON, but instead is quickly metabolized, potentially building up the pool of inorganic N instead. In the main text, Figure 6 and the discussion around it illustrate this phenomenon.

*Nonpoint Source-Based Yields: Models M1-M6*

Our first model, which we designate “M1” (Figure S1), simplifies the ELEMeNT conceptualization by combining all land uses into a single aggregate-watershed computational entity, and eliminating the protected SON pool from consideration (note that by omitting the recalcitrant SON pool from our models, we render their parameters not directly comparable with those of ELEMeNT). Eq. S3 simplifies to

$\frac{dM_{s}}{dt}=k_{a}M_{a}-\lambda_{s}M_{s}-J_{s}$	(S9)

As described by van Meter et al. (2017), when annual discharge (Q, m^3^/yr) is less than the source zone water volume (V_w_, m^3^), ELEMeNT treats J_s_ as directly proportional to discharge, in a fashion consistent with chemostasis (i.e., load proportional to flow) for the N source flux:

$J_{s}=\frac{Q}{V_{w}}M_{s}$	(S10)

We note that if V_w_ is assumed to be of fixed magnitude, then Q/V_w_ (i.e., inverse hydraulic residence time for the source zone) changes from year to year, directly with Q.

Normalizing both Q and V_w_ by watershed area, S10 can be expressed equivalently as:

$J_{s}=\frac{v}{L}M_{s}$	(S11)

where v is water yield, or discharge per watershed area (m/yr), and L (m) represents an effective depth for the SIN pool. Substituting S11 into S9 produces

$\frac{dM_{s}}{dt}=k_{a}M_{a}-\lambda_{s}M_{s}-\frac{v}{L}M_{s}$	(S12)

or equivalently in terms of J_s_:

$\frac{dJ_{s}}{dt}=\frac{v}{L}k_{a}M_{a}-\lambda_{s}J_{s}-\frac{v}{L}J_{s}$	(S13)

Within a given time step (i.e., year), while Q and therefore v/L is of fixed magnitude, S13 is equivalent to the mass balance of a completely mixed, stirred tank reactor (CSTR) for J_s_, given input N flux (kg ha^-1^ yr^-1^) of magnitude k_a_M_a_, and with first order denitrification and washout occurring at rates λ_s_ and v/L, respectively (yr^-1^). The general solution to eq. S13 for J_s_, with k_a_M_a_ treated as an initial condition, while v is of fixed magnitude, is

$J_{s}\left( t \right)=k_{a}M_{a}e^{-\left( \lambda_{s}+v/L \right)\left( t-t_{0} \right)}$	(S14)

Finally, riverine N yield (N_out, NPS_, kg ha^-1^ yr^-1^) resulting from nonpoint source inputs is calculated via convolution of the input source function J_s_ with the GIN (Groundwater Inorganic N) compartment’s TTD (Transit Time Distribution), f(τ), modified by first-order denitrification at rate γ (yr^-1^):

$N_{out, nps}=\int_{0}^{\infty} J_{s}\left( t-T \right)f\left( \tau\right)e^{-\gamma T}dT$	(S15)

Groundwater transit times are assumed in ELEMeNT to be exponentially distributed (van Meter et al., 2017), again consistent with a representation of the groundwater compartment as completely mixed:

$f\left( \tau\right)=\left( \frac{1}{\tau_{g}} \right)e^{-\left( \frac{1}{\tau_{g}} \right)T}$	(S16)

where τ_g_ is groundwater mean transit time. With denitrification incorporated, eq. S15 becomes

$N_{out,nps}=\int_{0}^{\infty} J_{s}\left( t-T \right)g\left( \tau\right)dT$	(S17)

where g(τ) is the IRF (Impulse Response Function, also sometimes referred to as a “transfer function”) for nitrate in the groundwater flow compartment (e.g., Howden et al., 2011):

$g\left( \tau\right)=\left( \frac{1}{\tau_{g}} \right)e^{-\left( \frac{1}{\tau_{g}}+\gamma\right)T}$	(S18)

Therefore, the nonpoint source contribution to riverine nitrate N yields is given by

$N_{out,nps}=\int_{0}^{\infty} J_{s}\left( t-T \right)\left( \frac{1}{\tau_{g}} \right)e^{-\left( \frac{1}{\tau_{g}}+\gamma\right)T}dT$	(S19)

Convolution of the N source function with an IRF of this form provides a means of expressing current-year N riverine yield as a sum of N inputs from the current year and all prior years, weighted in exponentially declining fashion with elapsed time since delivery from the source zone. The exponential nature of both the TTD and IRF means that irrespective of mean groundwater transit time, the greatest delivered fraction of annual yield originates from N input to the watershed in the same year. Although expressed in integral rather than differential form, S19 reflects the idea that the GIN compartment is, like the SIN pool, a completely mixed system (though one with stationary flow properties, unlike the SIN pool wherein flow changes from year to year), within which N is subject to first-order decay, and which is driven by annually varying input. In differential form, S19 can be expressed as:

$\frac{dN_{out,nps}}{dt}=\frac{1}{\tau_{g}}J_{s}-\left( \frac{1}{\tau_{g}}+\gamma\right)N_{out,nps}$	(S20)

The full set of equations for M1 can be summarized as

$\left\{ \begin{aligned} \frac{dM_{a}}{dt}=N_{surp}-k_{a}M_{a} \\ \frac{dM_{s}}{dt}=k_{a}M_{a}-\left( \frac{v}{L}+\lambda_{s} \right)M_{s} \\ J_{s}=\frac{v}{L}M_{s} \\ \frac{dN_{out,nps}}{dt}=\frac{1}{\tau_{g}}J_{s}-\left( \frac{1}{\tau_{g}}+\gamma\right)N_{out,nps} \end{aligned} \right.$	(S21)

Our second model, “M2” (Figure S2), is adapted from M1 to reflect the alternative conception that the SIN pool, rather than consisting of metabolized SON derived from accumulated present-plus-prior years’ N_surp_, derives solely from same year N_surp_:

$\left\{ \begin{aligned} \frac{dM_{s}}{dt}=N_{surp}-\left( \frac{v}{L}+\lambda_{s} \right)M_{s} \\ J_{s}=\frac{v}{L}M_{s} \\ \frac{dN_{out,nps}}{dt}=\frac{1}{\tau_{g}}J_{s}-\left( \frac{1}{\tau_{g}}+\gamma\right)N_{out,nps} \end{aligned} \right.$	(S22)

Importantly, when groundwater flow and reaction happen quickly compared with the time step magnitude (e.g., when τ_g_ <<1 year), eq. S20’s solution approaches that for steady-state:

$N_{out,nps}\cong\left( \frac{1}{1+\gamma\tau_{g}} \right)J_{s}$	(S23)

In such a circumstance, rather than meaningfully incorporating N from prior years into current-year riverine yield, convolution essentially just provides a scalar multiplier to translate same-year SIN flux from the soil root zone into riverine NO_3_-N yield. Because early calibrations of M1 and M2 revealed this to be the case at many sites, we developed two additional models that amount to simplifications of the M1 and M2 concepts by treating the SIN pool and GIN compartment as one conceptual/computational unit rather than two, essentially eliminating the GIN compartment as a discrete entity. Like model M1, model M3 (Figure S3) treats metabolized, accumulated SON as the input, and is therefore

$\left\{ \begin{aligned} \frac{dM_{a}}{dt}=N_{surp}-k_{a}M_{a} \\ \frac{dN_{out,nps}}{dt}=\frac{v}{L}k_{a}M_{a}-\left( \lambda_{s}+\frac{v}{L} \right)N_{out,nps} \end{aligned} \right.$	(S24)

Where N_out,nps_ has essentially replaced J_s_ in S13. Like M2, model M4 (Figure S4) treats N_surp_ rather than accumulated SON (i.e., M_a_) as the input, and is therefore

$\frac{dN_{out,nps}}{dt}=\frac{v}{L}N_{surp}-\left( \lambda_{s}+\frac{v}{L} \right)N_{out,nps}$	(S25)

In both M2 and M4, the yield adjustment embodied in S23 is accomplished largely through parameterization of the source N decay rate, λ_s_. Given the form of S25, the SIN pool in models M3 and M4 is essentially also a completely mixed reactor with first-order kinetics, though one with fluctuating residence times driven by annually varying flows as well as N inputs. Like the GIN compartment in models M1 and M2, in years when L/v is sufficiently small (e.g., << 1 year), the solution for the SIN pool in S24 approaches that of a steady-state (within time step) CSTR

$\left\{ \begin{aligned} \frac{dM_{a}}{dt}=N_{surp}-k_{a}M_{a} \\ N_{out,nps}=\left( \frac{1}{1+\frac{\lambda_{s}L}{v}} \right)k_{a}M_{a} \end{aligned} \right.$	(S26)

and eq. S25’s solution similarly approaches

$N_{out,nps}=\left( \frac{1}{1+\frac{\lambda_{s}L}{v}} \right)N_{surp}$	(S27)

We refer to S26 and S27 as models M5 (Figure S5) and M6 (Figure S6), respectively. M6 represents all nonpoint source contributions to annual riverine NO_3_-N yield in the simplest manner of any of the six models: as a function solely of same-year watershed net N input and riverine discharge. All processes that convert newly applied N in the watershed into riverine NO_3_-N within a time span of one year are embodied in the model’s single parameter, λ_s_L. This parameter thus encompasses aggregate TN transformation to NO_3_-N, partial loss to denitrification, and transport to surface water via all pathways, including direct overland runoff and movement through the subsurface.

In summary, for estimating nonpoint source based NO_3_-N riverine yields, we consider six contrasting models: three (M1, M3, M5) that treat SIN as originating from the breakdown of SON accumulated on the landscape over multiple years of anthropogenic inputs, and three (M2, M4, M6) that treat SIN as deriving solely from same-year N inputs (i.e., N_surp_). Models M3 and M4 are fully dynamic, while M6 can be thought of as reflecting pseudo steady-state conditions that are operative only within each annual time step. For M6, rather than λ_s_ and L being separate, tunable parameters that each influence NO_3_-N yield in different ways, only their product (λ_s_L) does so. In effect this product amounts to the watershed compartment’s sole parameter in models M5 and M6.

Note that like ELEMeNT, none of our models explicitly include an in-stream loss term for nonpoint-source derived N. Instead, such losses are subsumed within other, sometimes multiply redundant, model N loss terms (e.g., λ_s_, γ). As our models decline in mechanistic complexity going from M1 to M6, the remaining loss terms shoulder more of the burden of attenuating N loads. For M6, the λ_s_L/v construct effectively functions as a whole-system Damköhler number, with the watershed-plus-stream-network treated as if it were a single “reactor”.

*Point Source-Based Yields (Based on Human Population)*

The contributions from on-site disposal systems and wastewater-derived N loads to riverine N yields were approximated based upon estimates of total human nitrogen waste inputs to the watershed (e.g., Sabo et al., 2019; Byrnes et al., 2020a), adjusted to reflect all losses incurred during transport, including in-stream and/or in treatment plants. In-stream loss rates of N (as well as P) have been shown to vary inversely with stream depth and/or flow (e.g., Alexander et al., 2008; Alexander et al., 2009). Making use of established hydraulic geometry relationships between stream flow, depth, and velocity (Leopold and Maddock, 1953), Carleton and Mohamoud (2013) derived expressions for in-stream NO_3_ loss rates as power functions of these quantities, that seemed to represent observed N losses well, both empirically and when implemented in watershed model HSPF. Adopting the same general approach for point source-based N in this study, we chose to treat wastewater N loss coefficients as power functions of riverine flow, normalized by watershed area (i.e., v), and wastewater contributions to riverine N yield as governed by a within-year relationship similar in form to S27:

$N_{out, ps}=\left( \frac{N_{WWTP}}{1+Av^{B}} \right)$	(S31)

where N_WWTP_ is N inputs from human waste. Preliminary calibrations testing various other simple approaches (not shown) revealed this one to generally result in better overall model fits to data. In all six models, riverine NO_3_-N yields are calculated as the sum of yields derived from point sources and nonpoint sources:

$N_{out}=N_{out,nps}+N_{out, ps}$	(S32)

*Model Calibration*

The R scripts written to calibrate each model match the model predictions against corresponding WRTDS-generated annual riverine NO_3_-N yield time series at each site by maximizing Nash-Sutcliffe Efficiencies (NSE) – equivalent to minimizing summed squared deviations – between model output and WRTDS “Flux_K” output. The scripts run the models for the period 1930-2017, using WRTDS “Q” (annual riverine discharge), normalized by watershed area to calculate an effective annual mean watershed flow velocity, v (m/yr). For years that predate the WRTDS estimates (i.e.,1930-1971 or later), and that therefore lack tabulated Q values (relevant for all models that are capable of simulating delivery lag effects, i.e., all except M6), we substituted site-mean Q over the available range of years (≥1972-2017). The scripts employ functionality from the deSolve R package (Soetaert et al., 2010) to solve models M1 through M5 via numerical integration. The model M6 equation (S27) itself comprises an exact expression (thus no numerical approximation is necessary). Calibrations were conducted using either the “modFit” or “fminsearch” R functions from the FME package (Soetaert and Petzoldt, 2010) to search the parameter space for combinations that maximize NSE. We calculated AICc (Akaike Information Criteria adjusted for small sample size; Burnham and Anderson, 2003) scores from the summed squared errors associated with the optimized NSEs, to provide a metric for objective performance comparison between calibrated models that contain different numbers of adjustable parameters (Table 1).

**CITATIONS**

Alexander, R.B., Bӧhlke, J.K.B., Boyer, E.W., David, M.B., Harvey, J.W., Mulholland, P.J., Seitzinger, S.P., Tobias, C.R., Tonitto, C., & Wollheim, W.M. (2009). Dynamic modeling of nitrogen losses in river networks unravels the coupled effects of hydrological and biogeochemical processes. *Biogeochemistry*, 93:91-116.

Carleton, J.N., & Mohamoud, Y.M. (2013). Effect of flow depth and velocity on nitrate loss rates in natural channels. *Journal of the American Water Resources Association*, 49(1):205-216, doi:10.1111 ⁄ jawr.12007.

Leopold, L.B., & Maddock, T.M. (1953). The Hydraulic Geometry of Stream Channels and Some Physiographic Implications. USGS Professional Paper 252.

Liu, J., van Meter, K.J., McLeod, M.M., and Basu, N.B. (2021). Checkered landscapes: hydrologic and biogeochemical nitrogen legacies along the river continuum. Environmental Research letters, 66:115006, doi: 10.1088/1748-9326/ac243c.

Soetaert, K., & Petzoldt, T. (2010). Inverse modeling, sensitivity and Monte Carlo analysis in R sing package FME. *Journal of Statistical Software*, 33(3):1-28.

Soetaert, K., Petzoldt, T., & Setzer, R.W. (2010). Solving differential equations in R; package deSolve. *Journal of Statistical Software*, 33(9):1-25.

**Figure S1.** Schematic and equations of model M1.

**Figure S2.** Schematic and equations of model M2.

**Figure S3.** Schematic and equations of model M3.

**Figure S4.** Schematic and equations of model M4.

**Figure S5.** Schematic and equations of model M5.

**Figure S6.** Schematic and equations of model M6.

**Figure S7**. Cumulative distributions of ΔAICc values for M6 model calibrated using N_surp_(t) inputs lagged by from one to 20 years.

**Figure S8.** Boxplot showing distributions of coefficients of determination (R^2^) highlighting the strength of linear relationships between lagged N_surp_ values and same-year N_surp_ values across sites.

**Figure S9.** XY-plots showing the relationship between R^2^ from N_surp_ regressed against prior years’ N_surp_ vs. corresponding regression slopes. Blue dots represent lags of 1 to 10 years, and red dots represent lags of 11 to 20 years.

**Calibrated Model Time Series Figures** (see caption at end):

**Figure S10**. Calibrated NO_3_-N Yield time series plots for all 254 sites. Black lines represent NO_3_-N yields generated by the best-performing models (indicated on each subfigure), gray lines represent NO_3_-N yields generated by the other five models, and red circles represent “measured” (WRTDS-modeled) annual NO_3_-N yields. The best model’s name (i.e., M1 – M6) and associated NSE are annotated on each figure, as is the NSE range across all six models.

**Table S1**. Site watershed property summary.

|  | Watershed Area (km^2^) | Total Percent Forest* | Total Percent Urban* | Total Percent Agriculture* |
| --- | --- | --- | --- | --- |
| Range | 9.9 – 2,878,560.8 | 0.21 – 100 | 0 – 82.5 | 0 – 91.1 |
| Mean | 64,330.3 | 37.0 | 7.8 | 27.5 |
| Median | 2,041.5 | 36.5 | 3.0 | 22.2 |
| Std. Dev. | 303,826.9 | 24.2 | 13.2 | 25.2 |

*Based on 2011 NLCD.

**Table S2**. Site watershed properties (mean ± standard deviation) by highest-quality model.

| Best model | Watershed Area (km^2^) | Percent Imperviousness | Total Percent Urban | Total Percent Agriculture | Total Percent Forest | Mean WWTP Percent of Total N Input |
| --- | --- | --- | --- | --- | --- | --- |
| M1 | 8,516 ± 12,826 | 3.7 ± 3.3 | 6.6 ± 5.9 | 20.6 ± 16.9 | 44.5 ± 17.1 | 10.9 ± 11.5 |
| M2 | 9,085 ± 13,973 | 6.6 ± 12.2 | 11.6 ± 21.0 | 17.1 ± 13.8 | 46.3 ± 23.0 | 12.4 ± 10.7 |
| M3 | 164,117 ± 435,491 | 2.8 ± 5.8 | 4.9 ± 10.1 | 22.6 ± 20.2 | 21.1 ± 19.5 | 9.6 ± 16.8 |
| M4 | 14,129 ± 19,934 | 2.3 ± 3.2 | 4.1 ± 5.7 | 31.1 ± 31.5 | 30.7 ± 32.1 | 9.6 ± 12.5 |
| M5 | 85,551 ± 246,626 | 2.1 ± 4.0 | 3.8 ± 7.4 | 29.2 ± 27.3 | 41.0 ± 26.6 | 5.9 ± 11.3 |
| M6 | 55,717 ± 331,867 | 5.3 ± 8.4 | 9.6 ± 14.7 | 28.1 ± 25.2 | 36.9 ± 22.9 | 14.3 ± 16.7 |

**Table S3**. Parameters of six calibrated models for six example sites shown in Figure 4. Blank cells indicate parameters that were not included in specific models. Italicized λ_s_L values were calculated from separately calibrated λ_s_ and L parameters in models M1 - M4.

| Site | Model | k_a_ | λ_s_ | L | λ_s_L* | τ_g_ | γ | A | B |
| --- | --- | --- | --- | --- | --- | --- | --- | --- | --- |
| Arkansas River | M1 | 58.6 | 14.5 | 0.001 | *0.02* | 8.0 | 6.0 | 0.1 | -1.0 |
|  | M2 |  | 15.3 | 0.001 | *0.02* | 3.6 | 13.1 | 0.1 | -1.0 |
|  | M3 | 138.8 | 7.0 | 0.1 | *0.7* |  |  | 120.0 | 0.0 |
|  | M4 |  | 10.0 | 0.1 | *0.7* |  |  | 2.0 | -0.6 |
|  | M5 | 437.7 |  |  | 1.5 |  |  | 0.1 | -0.9 |
|  | M6 |  |  |  | 0.7 |  |  | 9.8 | -0.4 |
| Ocmulgee River | M1 | 42.0 | 0.0 | 2.1 | *0.0* | 1.0 | 28.0 | 24.2 | 0.3 |
|  | M2 |  | 29.8 | 0.001 | *0.0* | 1.6 | 19.9 | 3.7 | -0.9 |
|  | M3 | 0.1 | 5.3 | 1.9 | *10.0* |  |  | 11.0 | -0.1 |
|  | M4 |  | 9.6 | 1.4 | *13.6* |  |  | 6.6 | -0.2 |
|  | M5 | 0.2 |  |  | 11.5 |  |  | 10.7 | 0.0 |
|  | M6 |  |  |  | 13.7 |  |  | 7.4 | -0.1 |
| Saddle River | M1 | 127.2 | 0.0 | 3.4 | *0.0* | 1.3 | 2.9 | 2.2 | -0.5 |
|  | M2 |  | 0.0 | 3.9 | *0.0* | 1.0 | 2.9 | 2.6 | -0.5 |
|  | M3 | 0.1 | 8.1 | 0.3 | *2.7* |  |  | 6.4 | -0.9 |
|  | M4 |  | 10.0 | 0.3 | *2.9* |  |  | 2.1 | -0.6 |
|  | M5 | 0.02 |  |  | 0.1 |  |  | 4.8 | -0.9 |
|  | M6 |  |  |  | 3.2 |  |  | 2.0 | -0.6 |
| Susquehanna River | M1 | 99.5 | 0.0 | 3.2 | *0.0* | 1.0 | 4.9 | 1.8 | -1.0 |
|  | M2 |  | 0.0 | 2.9 | *0.0* | 1.0 | 5.0 | 2.1 | -1.0 |
|  | M3 | 0.4 | 200.0 | 0.01 | *2.3* |  |  | 4.3 | -1.0 |
|  | M4 |  | 20.2 | 0.1 | *2.4* |  |  | 1.7 | -1.0 |
|  | M5 | 0.4 |  |  | 2.3 |  |  | 3.3 | -1.0 |
|  | M6 |  |  |  | 2.5 |  |  | 1.6 | -1.0 |
| Trinity River | M1 | 0.5 | 7.5 | 0.003 | 0.0 | 1.5 | 9.3 | 2.4 | -0.7 |
|  | M2 |  | 44.5 | 0.001 | 0.0 | 1.0 | 13.5 | 2.8 | -0.4 |
|  | M3 | 0.6 | 10.4 | 0.4 | 3.8 |  |  | 5.1 | 0.1 |
|  | M4 |  | 9.9 | 0.4 | *4.0* |  |  | 4.5 | 0.0 |
|  | M5 | 0.9 |  |  | 4.3 |  |  | 5.3 | 0.1 |
|  | M6 |  |  |  | 4.5 |  |  | 4.8 | 0.1 |
| Willamette River | M1 | 36.8 | 0.0 | 3.9 | *0.0* | 1.0 | 3.6 | 4.2 | -1.0 |
|  | M2 |  | 0.002 | 3.9 | *0.006* | 1.0 | 3.4 | 10.9 | -1.0 |
|  | M3 | 110.0 | 321.3 | 0.01 | *3.5* |  |  | 13.0 | -1.0 |
|  | M4 |  | 65.7 | 0.1 | *3.4* |  |  | 80.4 | -1.0 |
|  | M5 | 160.0 |  |  | 3.5 |  |  | 14.2 | -1.0 |
|  | M6 |  |  |  | 3.5 |  |  | 10.5 | -1.0 |
